# Familial Mediterranean fever; diagnosis, treatment, and complications

**Published:** 2015-01-01

**Authors:** Bahman Bashardoust

**Affiliations:** ^1^Department of Internal Medicine, Ardabil University of Medical Sciences, Ardabil, Iran

**Keywords:** Familial Mediterranean fever, Renal

**Implication for health policy/practice/research/medical education:**

Mediterranean fever is an autosomal recessive disease. Its features are intermittent attacks of painful inflammation, abdominal pain, fever, and arthritis. Its attacks take from a few hours to a few days of symptoms and the recurrence takes a few weeks or months.

## Introduction

Mediterranean fever is an autosomal recessive disease. Its features are intermittent attacks of painful inflammation, abdominal pain, fever, and arthritis. Full identification of the disease has been possible in the last 50 years. It is seen in Turkish, Armenian, Jewish (Arabs, Ashkenazi) and Mediterranean region ethnics. Its attacks take from a few hours to a few days of symptoms and the recurrence takes a few weeks or months. Salehzadeh and colleagues reported the first comprehensive Mediterranean fever patient in Ardabil region (1).

## Diagnosis

The diagnosis of Familial Mediterranean fever (FMF) is based on Tel-Hashomer clinical criteria, which is two or more major symptoms or one major plus two minor symptom. Major and minor Tel-Hashomer clinical criteria are presented in Table 1 (2).

**Table 1:** Tel-Hashomer diagnosis criteria

**Major criteria**	**Minor criteria**
Recurrent febrile episodes with serositis (peritonitis, synovitis or pleuritis)	Recurrent febrile episodes
Amyloidosis of AA type without a predisposing disease	Erysipelas-like erythema
Favorable response to regular colchicine treatment	FMF in a first-degree relative

Livneh *et al.* suggested that the diagnostic criteria includes typical, incomplete, and supportive. A simplified version of it is given in Table 2 (3).

**Table 2:** Simplified FMF diagnosis criteria suggested by Livneh *et al.*

**Major criteria**	**Minor criteria**
Typical attacks (1-4)	Incomplete attacks involving either or both of the following sites
1- Generalized peritonitis	1- Chest
2- Unilateral pleuritis or pericarditis	2- Joint
3- Monoarthritis (hip, knee, ankle)	3- Exertional leg pain
4- Fever alone	4- favorable response to colchicine
5- Incomplete abdominal attack	

The requirements for the diagnosis of FMF have been defined as the presence of: at least one major; or at least two minor criteria. Typical attacks must include all the following: recurrent (at least three episodes), febrile (rectal temperature ≥ 38 °C) and short in duration (12 hours to 3 days). Incomplete attacks (must be recurrent) are defined as differing from typical attacks in one or two features as follows: 1) temperature <38 °C, 2) attack duration longer or shorter than a typical attack (but no less than six hours and no more than seven days), 3) no signs of peritonitis during the attacks, 4) localized abdominal attacks, and finally 5) arthritis in a location other than the hip, knee or ankle.

Mediterranean fever genetic testing can be used to detect at least two heterozygote mutations or a homozygous mutation that is necessary.

## Treatment

Targeted therapy to treat acute attacks, prevent relapses and is suppressed by chronic inflammation and prevent complications. Unfortunately, the exact evaluation of treatment response, such as systemic lupus erythematosus, is not available. Mediterranean fever can be diagnosed wrongly with many organic and non-organic diseases. Also a high incidence of psychosomatic and fibromyalgia disease can make diagnosis and treatment more and more difficult.

Colchicine has been well-known since 1970 that uses its impact with 1 mg per day, if it does not respond to treatment it can be raised to 1.5 to 2 mg/day, and it can be administered twice a day if is not tolerated as a single dose (4-6).

Studies of resistance to colchicine have been reported in 5% to 10% (7-9). Hence, if a patient is being treated with colchicine, other circumstances should be considered too, especially if the patient, has previously received treatment for other diseases. Using 40 mg of intravenous methylprednisolone (10) in acute phase, alpha interferon, IL-1 cytokine antagonist (11), IL1 alpha and IL-1 beta blocker (12,13), anti TNF (14) and dapsone (15) is reported to be effective.

## Complications

**Figure 1:**
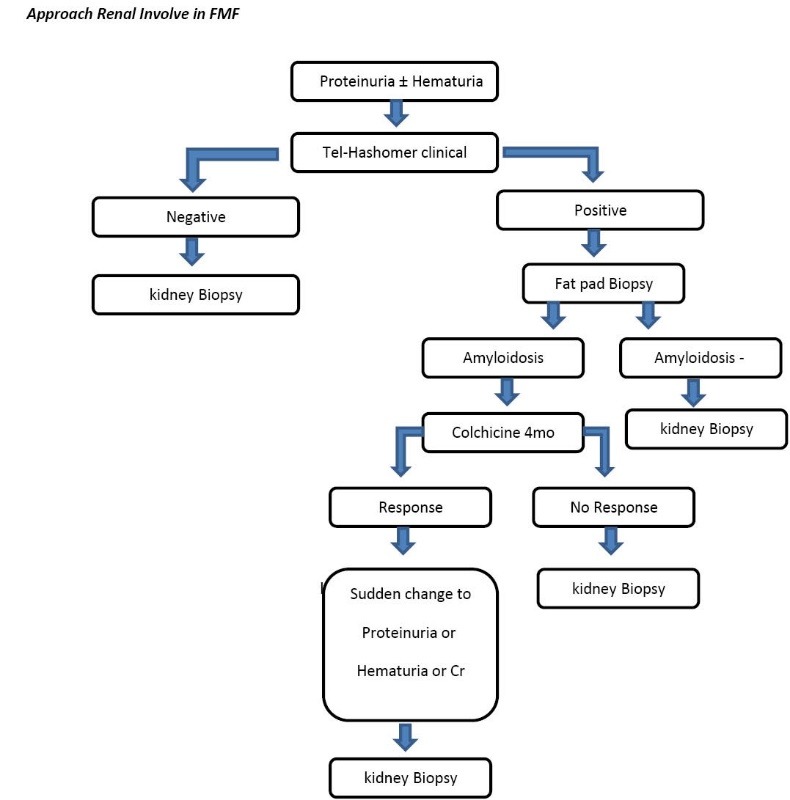
Approach Renal Involve in FMF

Amyloidosis is the most common complication of FMF (16), and it determines whether the prognosis of the disease is associated with progression to end-stage renal disease (10-16). Colchicine prevents the occurrence of amyloidosis, to stop amyloidosis, and even regress it. The duration of the disease is not the main cause of amyloidosis but specific genetic and environmental conditions is necessary. Prevalence of amyloidosis in Armenians is 24% but about those Armenians living in California no amyloidosis has been reported (17). Homozygote of M694V is mostly observed in Amyloidosis patients (18). End-stage renal disease and nephrotic syndrome is the most common finding in amyloidosis. Mediterranean fever patients need to be evaluated against proteinuria regularly (15-18). Some studies on fat skin biopsy for amyloidosis have low sensitivity in evaluations (19-21). We have shown that the beneath fat pad skin biopsy is valuable and reduces the need for renal biopsy (Figure 1) (22). Peritoneal mesothelioma due to chronic inflammation has been reported (23). Hip involvement is destructive in which ultimately needs to have joint replacement (24,25). Ankylosing spondylitis is a form of spinal involvement and has no relation with HLA B27 (26-28). Early atherosclerosis that is similar to some patients with rheumatic diseases has been seen. Also, valvular involvement particularly aortic is observed (29,30). Involvement of conducting system of the heart and amyloidosis arthropathy are also other complications of FMF. PAN (31), Henoch-Schonlein purpura and Behcet’s syndrome (30-33) in FMF patients has high incidence. Among our patients the main cause of chronic kidney disease was focal segmental glomerulosclerosis (22).

## Author’s Contribution

BB was the single author of the paper.

## Conflict of interests

None to declare.

## Ethical considerations

Ethical issues (including plagiarism, misconduct, data fabrication, falsification, double publication or submission, redundancy) have been completely observed by the author.
